# USING VIRTUAL REALITY IN AGED CARE SETTINGS: A SCOPING REVIEW

**DOI:** 10.1093/geroni/igac059.2850

**Published:** 2022-12-20

**Authors:** Lillian Hung, Flora To-Miles, Winnie Kan, Alisha Temirova, Jim Mann, Christine Wallsworth

**Affiliations:** University of British Columbia, Vancouver, British Columbia, Canada; University of British Columbia, Vancouver, British Columbia, Canada; University of British Columbia, Vancouver, British Columbia, Canada; University of British Columbia, Vancouver, British Columbia, Canada; Community Engagement Advisory Network, Vancouver, British Columbia, Canada; Vancouver, British Columbia, Canada

## Abstract

Recent advancement of virtual reality (VR) technology has led to growing interest in using VR in aged-care settings. VR can help ameliorate experiences of loneliness and social isolation, which is especially important during the COVID-19 pandemic. As a result, increasingly more studies are being published on this topic, and a comprehensive review of studies examining the facilitators and barriers of adopting VR in these settings is needed. This scoping review reports the facilitators and barriers to implementing VR in care settings among older adults, as well as the impact on social engagement and/or loneliness. We followed the Joanna Briggs Institute scoping review methodology, and searched the following databases: CINHAL, Embase, Medline, PsycInfo, Scopus, and Web of Science. Inclusion criteria includes articles published in the last five years that focus on older adults using VR in aged-care settings. 199 articles were retrieved and 21 articles were included in our review. Most of the articles (38%) originated from Australia. Key facilitators for using VR in aged care settings are the technology being user-friendly, comfortable, and easy to clean. Barriers included: technology issues (e.g., internet connectivity), staff attitude/ worries, and impact on residents, and structural considerations (e.g., lack of staff and time to assist with the VR program). VR technology can decrease loneliness and feelings of isolation, and provide opportunities to engage with others. Our review of the current evidence offers insights and recommendations for health care professionals to use VR technology in aged care settings, maximizing benefits and minimizing risks among users.

